# Supervised extensions of chemography approaches: case studies of chemical liabilities assessment

**DOI:** 10.1186/1758-2946-6-20

**Published:** 2014-05-07

**Authors:** Svetlana I Ovchinnikova, Arseniy A Bykov, Aslan Yu Tsivadze, Evgeny P Dyachkov, Natalia V Kireeva

**Affiliations:** 1 Frumkin Institute of Physical Chemistry and Electrochemistry RAS, Leninsky pr-t 31-4, 119071 Moscow, Russia; 2 Moscow Institute of Physics and Technology, Institutsky per., 9, 141700 Dolgoprudny, Russia; 3 Kurnakov Institute of General and Inorganic Chemistry RAS, Leninsky pr-t 31, 119071 Moscow, Russia

**Keywords:** Cheminformatics, Chemography, Applicability domain, Generative topographic mapping, Dimensionality reduction, Supervised generative topographic mapping, Isomap, Supervised Isomap

## Abstract

Chemical liabilities, such as adverse effects and toxicity, play a significant role in modern drug discovery process. *In silico* assessment of chemical liabilities is an important step aimed to reduce costs and animal testing by complementing or replacing *in vitro* and *in vivo* experiments. Herein, we propose an approach combining several classification and chemography methods to be able to predict chemical liabilities and to interpret obtained results in the context of impact of structural changes of compounds on their pharmacological profile. To our knowledge for the first time, the supervised extension of Generative Topographic Mapping is proposed as an effective new chemography method. New approach for mapping new data using supervised Isomap without re-building models from the scratch has been proposed. Two approaches for estimation of model’s applicability domain are used in our study to our knowledge for the first time in chemoinformatics. The structural alerts responsible for the negative characteristics of pharmacological profile of chemical compounds has been found as a result of model interpretation.

## Background

During the past decade, computational technologies and predictive tools have been deeply integrated in the modern drug discovery process and changed this process extracting the useful knowledge embedded in the complex arrays of chemical and biological information to select the most promising compounds as early as possible and to reveal chemical liabilities in order to reduce the risk of late stage attrition [1,2]. Chemical liabilities, such as adverse effects and toxicity, play a significant role in modern drug discovery process. Methods to avoid or reduce chemical liabilities are an important target for drug discovery and development. Herein, we propose an approach combining several classification and chemography [3] methods to assess chemical liabilities *in silico* and to interpret obtained results in the context of impact of structural changes of compounds on their pharmacological profile. Model development has been performed in six different descriptor spaces for mutagenicity, carcinogenicity, acute toxicity and phospholipidosis data sets. A set of machine learning methods has been involved in model development encompassing well-known approaches with new ones. The combination of classification and data visualization is a key point for mechanistic model interpretation which allows one to understand which changes of the existing structures are required to improve target properties, to generate new hypothesis and, finally, to optimize the chemical structures. Over the years, a number of dimensionality reduction approaches [4-11] have been proposed and used in cheminformatics. The most known and widely used among these methods are Principal Component Analysis [12], Multidimensional Scaling (MDS) [13,14], Self-Organizing Maps (SOM) [15], Stochastic Proximity Embedding [16-18], Stochastic Neighbor Embedding [19,20], Sammon Mapping [21] and Generative Topographic Mapping (GTM) [22-24]. In this study, Generative Topographic Mapping and Isomap as well as their supervised extensions have been involved. Recently, the unsupervised implementations of these approaches have been used in a number of studies in chemoinformatics [25-32]. These two representatives of nonlinear dimensionality reduction methods are related to two different families: distance-based approaches and topology based approaches. Isomap reduces the dimensionality of data by using distance preservation as the criterion, that is intuitively understandable and easy to compute. GTM is related to the topology based techniques. This group of methods tries to preserve topology principle that is concerned to relative proximities: compounds which are close in the data space remain close in the data visualization model. Topology preservation usually is considered as more powerful and in the same time more complex comparing with distance preservation [4]. The comparison of used techniques on the considered data is performed in this study. Support vector machines (SVM) [33], GTM and probabilistic neural networks (PNN) [34] have been used for the development of classification models. Two applicability domain of models’ approaches (AD) are involved in our study in order to assesses the model’s limitation in prediction of new data in order to reliably predict those data that are structurally similar to the training set compounds used for model development. Recently, several different AD approaches have been proposed [35-49]. Here, we use the representatives of two families of AD methods: distance-based (Ball) [50] and probability-based (Local Outlier Factor LOF) [51].

Here, to our knowledge for the first time, we propose supervised extension of Generative Topographic Maps [52] that can be used as a universal tool to visualize the chemical space and to develop classification models. New approach for projecting new data using supervised Isomap [53] without re-building models from the scratch has been developed. The evaluation of the performance of the dimensionality reduction techniques and introduced descriptor spaces to separate different activity classes has been monitored by three parameters, two of them have been used in cheminformatics for the first time.

## Materials and methods

### Data preparation

Data preparation has been carried out using recommendations published in [54]. Chemaxon Standardizer [55] and Instant JChem [56] software have been used for the data preparation. Using Standardizer, the explicit hydrogen atoms have been removed, the structures have been aromatized and neutralized. Four data sets have been used in our study.

#### ***Mutagenicity***

Ames mutagenicity data from a study by Kazius et al. [57]. The data set contained 2367 active and 1888 inactive compounds. External test set consists of 1164 active and 2167 inactive compounds.

#### ***Carcinogenicity***

Data was collected from the distributed ISSCAN Database (part of structure-searchable toxicity DSSTox public database network [58]). The database has been specifically designed as an expert decision support tool and includes the carcinogenicity classification “calls” to guide the application of SAR approaches. Collected data set encompass 1088 chemical structures containing 648 compounds annotated as actives and 440 as inactive compounds. External test set [25] contains 359 actives and 141 inactives.

#### ***Phospholipidosis***

A set of 100 phospholipidosis-inducing compounds and 82 negative drug-like compounds were taken from [59], where the active compounds have been observed to act on a range of species (humans, rats, mice, dogs, rabbits, hamsters and monkeys) and on a variety of tissue types (lungs, kidney and liver). External test set from [60] contains 141 active and 359 inactive compounds.

#### ***Acute toxicity***

Data from EPA Fathead Minnow Acute Toxicity Database [61] after data preparation stage containing 612 compounds (578 actives and 34 inactives). This database was generated by the U.S. EPA Mid-Continental Ecology Division (MED) for the purpose of developing an expert system to predict acute toxicity from chemical structures based on mode of action considerations.

### Descriptors

In this study, six descriptor types have been involved in model development. ISIDA package [62] has been represented by two different descriptor types: ***(i)*** ISIDA Property-Labeled Fragment Descriptors (IPLF) [63] (atom-centered fragments (augmented atoms) of radius 1 to 3 colored by pH-dependent pharmacophores and ***(ii)*** subclass of ISIDA Substructural Molecular Fragments (SMF) [62] consisting of the shortest topological paths with explicit representation of only terminal atoms and bonds, where the values of minimal *n*_*min*_ and maximal *n*_*max*_ number of atoms varied from 2 to 15. 2D descriptors of Molecular Operating Environment (MOE 2D) [64] containing different physical properties, subdivided surface areas, atom and bond counts, Kier&Hall connectivity and Kappa shape indices, adjacency and distance matrix descriptors, pharmacophore feature descriptors and partial charge descriptors were involved in model development. The CDK (Chemistry Development Kit) MACCS keys and extended fingerprints (EF) were computed using the RCDK package [65] of the R software [66]. Finally, Dragon software [67] has been used for molecular descriptors calculations. Constant and nearly constant descriptors were removed. Detailed table with the final number of descriptors for each data set and descriptor type is represented in supporting information.

## Methods

### Classification methods

#### ***Support Vector Machines (SVM)***

SVM [68,69] is a supervised learning method commonly used for classification and regression and based on statistical learning theory of Vapnik–Chervonenkis [70,71]. Projecting the original data described by means of descriptor vectors to a higher dimensional feature space SVM achieves distinct separation between considered classes of compounds finding the optimal position of the separating hyperplane between the instances from the classes.

#### ***Generative Topographic Mapping (GTM)***

GTM is a specific unsupervised density network based on generative modeling. It can be considered as probabilistic extension of Kohonen Self-Organizing Maps. Like SOM, it operates with a grid of K nodes, which can be considered as analogs of nodes in SOM. GTM creates a generative probabilistic model in the high-dimensional data space RD by placing a radially symmetric Gaussian with zero mean and inverse variance *β* around projections of the latent space centers which approximating the data density. The nonlinear GTM transformation from the latent space to the data space is defined using a Radial Basis Function (RBF) network. Thus, each node is projected to the center of Gaussian belonging to the manifold (two-dimensional flexible sheet located in the high-dimensional space in such a way to cover the data points by stretching or compressing) embedded in the data space. This manifold can be considered as a representation of the latent space in the data space. The coordinates of the Gaussians are computed as a linear combination of Gaussian basis functions and for the point ***x*** in the latent space its projection to the data space can be defined as:

(1) $$y=W\phi \left(x\right)$$

where W- the output weights of RBF.

It relates the real data in the chemical space with manifold points. Thus, any point of the latent space ℝ^*L*^ has its own projection in a data space ℝ^*D*^ obtained by non-linear parameterized mapping *y*(***x***, W).

The mapping function *y*(***x***, W) is continuous, which leads to the topographic ordering of the projected points, i.e. two points that are close in the latent space are also close in the data space. Defining a probability distribution over the latent space induces the corresponding distribution over the manifold in the data space and, thus, imposes the probabilistic relationships between two spaces.

The iterative Expectation-Maximization algorithm (EM-algorithm) is used to find the parameters of RBF network (W and *β*) maximizing the, so called, log likelihood function which measures a correspondence between the data distribution and the model.

(2) $$ℒ\left(W,\beta \right)=\overset{N}{\underset{n=1}{\sum }}\ln\left\{\frac{1}{K}\overset{K}{\underset{i=1}{\sum }}p\left(t_{n}\left|x_{i},W,\beta \right)\right)\right\}$$

where ℒ - log likelihood function, *β* - inverse of variance, W - the output weights of RBF, *K* - number of the nodes, *N* - number of compounds, *p*(***t***_***n***_| ***x***_***i***_, W, *β*) – prior probability generated in a point ***t***_*n*_ in the data space by the Gaussian with a center in *y*(***x***_*i*_, W).

Activity profile of a chemical compound can be assessed starting from the values of the class-conditioned probability distribution function *p*(***t*** |*C*_*k*_) computed for each class *C*_*k*_, where ***t*** is its molecular descriptor vector. Such function can be build, for each activity class, by training a separate GTM model on the data belonging to class *C*_*k*_. The class-conditioned probabilities *p*(***t*** |*C*_*k*_) can be used for computing posterior probabilities of class membership *P*(*C*_*k*_| ***t***) for a given compound using the Bayes theorem:

(3) $$P\left(C_{k}\left|t\right)\right)=\frac{p\left(t\left|C_{k}\right)\right)\cdot P\left(C_{k}\right)}{p\left(t\right)}$$

where $P\left(C_{k}\right)=\frac{N_{k}}{N_{\text{tot}}}$ is a prior probability of class membership (*N*_*k*_ – the number of compounds belonging to class *C*_*k*_; *N*_*tot*_*–* the total number of compounds), whereas *p*(***t***), the marginal probability density function, is the normalization factor:

(4) $$p\left(t\right)=\underset{k}{\sum }p\left(t\left|C_{k}\right)\right)\cdot P\left(C_{k}\right)$$

The latter ensures that the estimated posterior probabilities are normalized. By applying Function 3 to each class *C*_*k*_ one can assess the posterior probability of class membership for each compound. According to statistical decision theory [72], the optimal class assignment is determined by the maximal value of posterior class probabilities *P*(*C*_*k*_| ***t***).

#### ***Probabilistic Neural Networks (PNN)***

PNN [34] belongs to a group of feed-forward neural network algorithms. It was derived from Bayesian Networks [73] and Kernel Discriminant Analysis [74]. PNN consists of four layers: input layer, pattern layer, summation layer and output layer.

An input layer represents the input vector, e.g. a compound from a test set. Each compound is attributed to a single neuron of pattern layer, for which its descriptors represent a weight vector. Therefore all pattern neurons can be marked with the class labels of corresponding compounds. Input layer interconnected with a pattern layer, thus each pattern unit forms a dot product *Z* of an input vector and its weight vector. *Z* is propagated to the network activation function $e^{\frac{Z-1}{\sigma^{2}}}$ and the result is outputted to the summation layer. Each neuron in the summation layer is connected to pattern units of the corresponding class. This layer performs simple summation of the inputs from the pattern layer. The output layer is a two-input layer, which produces a binary output. It takes into account the contribution for each class of inputs. The output is a 1 (positive identification) for that class and a 0 (negative identification) for non-targeted classes. In fact, there’s no training required since the compounds of the training set are considered as the weights to the hidden layer of the network. As no training required, classifying an input vector is fast, depending on the number of classes and compounds in use. PNNs have some advantages comparing with multilayer perceptron networks: they are faster, relatively insensitive to outliers and generate probability scores.

### Dimensionality reduction methods

#### ***Supervised Generative Topographic Mapping (s-GTM)***

GTM performs visualization by inversing mapping from the data space to the latent space (unbending this flexible sheet into the rectangular 2D map). For this Bayes theorem is used. Thus, for each molecule GTM calculates its probability to be located in the given point of this map represented by the latent space and visualizes this molecule according this probability.

In order to make manifolds location in the data space dependent on distribution not only of the whole data set, but also of each class, a new supervised training procedure was performed. Each iteration consists of two major steps.

On the first step latent points are ascribed to one of the data classes in consideration. To this end we calculate responsibilities *r*_*kn*_ (i.e. posterior probabilities that data point ***t***_*n*_ was generated by the component ***x***_*k*_).

(5) $$r_{\mathrm{kn}}=p\left(x_{k}\left|t_{n},W,\beta \right)\right)=\frac{p\left(t_{n}\left|x_{k},W,\beta \right)\right)p\left(x_{k}\right)}{\sum_{i=1}^{K}p\left(t_{n}\left|x_{i},W,\beta \right)\right)p\left(x_{i}\right)}$$

where $p\left(x_{k}\right)=\frac{1}{K}$.

For each latent point ***x***_*k*_ the following sums are calculated

(6) $$S_{j}=\frac{1}{N_{j}}\overset{N_{j}}{\underset{i=1}{\sum }}r_{\mathrm{ki}},j=1,2$$

where index *j* refers to one of the classes, *N*_*j*_ – is a number of compounds in this class.

The latent point is associated with the class with the largest sum of responsibilities, only in a case when the difference between the sums is greater than the threshold value *thr*, which is an external parameter of the method. If not, the latent point remains unlabeled. To assure the formation of clusters of similarly labeled latent points, the influence of neighbor latent points is taken into account by decreasing the threshold value if the latent point on previous iteration had neighbors associated with the class which responsibility sum is larger on the current iteration and increasing it if the neighbors are from the opposite class.

The second step contains movement of the latent points projections towards data points of the corresponding class by adjusting the RBF network. The sum $\overset{¯}{T}$ of vector distances from the latent point ***x***_*k*_ projection to all the data points ***t***_*n*_, for which *r*_*kn*_ > *rr*, is calculated. Here, *rr* denotes the responsibility radius, another external parameter of s-GTM. If the data point belongs to the class opposite to that of the latent point, the corresponding distance is multiplied by -1 (thereby, the vector form the data point to the latent point is obtained). The desired new coordinates P¯' of the latent point projection are defined the following way:

(7) P¯'=P¯+T¯N

Then RBF network is trained using the coordinates of ***x***_*k*_ in the latent space as input and P¯' as a target.

Supervised GTM has a number of external parameters that have a great influence on the model development. Main parameter for latent points’ colorization is the threshold value. It should be low enough, in order to allow a considerable amount of latent points to get labeled. The maximum value can be found from analyzing the responsibility matrix and strongly depends on the number of latent points: the larger is their number, the lower should be the threshold value.

The influence of the color of neighbor latent point is defined by additional compound for threshold calculation:

(8) $$\text{thr}{'}_{1,l}=\text{thr}+\frac{N_{2}-N_{1}}{\rho }$$

where *thr* is an original value, *N*_1_ and *N*_2_ – number of neighboring latent points of class 1 and 2 respectively, *ρ* - an external parameter, *thr* '_1,*l*_ - is a threshold value, specific for class 1 and latent point *l*. This means, that latent point *l* will be labeled as class 1, if

(9) $$S_{1}>S_{2}+\text{thr}{'}_{1,l}$$

It is obvious, that parameter *ρ* is required to bring both terms of Formula 9 to similar scale. Surprisingly, in quite a wide range it has small impact on the model, but can be very useful for imbalanced data to prevent all the latent point to be marked by the same class label. It should be altered for fine optimization or in case if no similarly labeled clusters of latent points are formed during the training process.

#### ***S-Isomap***

Isomap [75] is a low-dimensional embedding method. It implies that data are disposed along a manifold with a dimensionality *d* less than dimensionality *d*_*o*_ of the original data space. Our aim is to “unroll” the manifold into a d-dimensional space, so that data points, which are close to each other on the manifold remain close, and remote points – stay remote. To this end, we replace Euclidian distance with geodesic one – the length of the shortest curve between two points that lies on the manifold.

Isomap algorithm consists of three steps. On the first step we define *k* nearest neighbors of each compound and assume that Euclidian distances between them are small and, thus, are nearly equal to corresponding geodesic distances. This assumption allows us to create a weighted graph where only the vertices that are nearest neighbors are connected and the length of each edge equals the corresponding distance. This graph is not always connected and in this case the largest connected part is taken for the next step. After the graph has been constructed we compute shortest distances between its vertices. Then obtained distance matrix is used for multidimensional scaling (MDS) [13,14] from original to *d*-dimensional space. To minimize the cost function in MDS coordinates of compounds in the new space should be set to the top *d* eigenvectors of the matrix $\tau \left(\overset{˜}{D}\right)$[76], where $\overset{˜}{D}$ is a matrix of pairwise distances between training points and *τ* is an operator, that converts distances to inner products. For visualization purpose we set *d* = 2.

Supervised extension of Isomap was proposed in [53]. It differs from the original algorithm in its first step. Instead of Euclidian distance *d*(***x***_*i*_*,* ***x***_*j*_) between ***x***_*i*_ and ***x***_*j*_ a new measurement of compounds’ dissimilarity is calculated.

(10) $$D\left(x_{i},x_{i}\right)=\left\{\begin{array}{c} \sqrt{1-e^{\frac{-d^{2}\left(x_{i},x_{j}\right)}{\beta }}},\mathrm{if}\;y_{i}=y_{j}\\ \sqrt{e^{\frac{d^{2}\left(x_{i},x_{j}\right)}{\beta }}}-\alpha ,\mathrm{if}\;y_{i}\neq y_{j} \end{array}\right)$$

Here *y*_*i*_ denotes class label of compound ***x***_*i*_, and *β* is a parameter that prevents *D*(***x***_*i*_, ***x***_*j*_) from increasing too fast. *β* should depend on data density and average Euclidian distance between all pairs of data points is usually used. The parameter *α* gives some chance to the points from different classes to be more close to each other.

After new distances have been calculated k-nearest neighbors are defined and weighted graph is constructed in the way it is done in non-supervised algorithm.

A way to extend nonsupervised Isomap to new points was proposed in [77,78]. There coordinates of new points are calculated as

(11) $$e_{k}\left(x\right)=\frac{1}{2\sqrt{\lambda_{k}}}\underset{i}{\sum }\upsilon_{\mathrm{ki}}\left(E_{x^{'}}\left(\tau \left(\overset{˜}{D}\left(x^{'},x_{i}\right)\right)\right)-\tau \left(\overset{˜}{D}\left(x_{i},x\right)\right)\right)$$

where *λ*_*k*_ is eigenvalues and *υ*_*ki*_ - coordinates of the corresponding eigenvectors of the matrix $\tau \left(\overset{˜}{D}\right)$, operator $E_{x^{'}}$ denotes average over the data set. To make this work for S-Isomap we take into consideration Eq. 11, while computing $\overset{˜}{D}\left(x_{i},x\right)$ – geodesic distance from and external point ***x*** to the training point ***x***_*i*_. We assume that the distance from ***x*** to its ***k*** nearest neighbors of ***x*** is small enough to make not much difference between two parts of Eq. 11, and so we can use their average as a geodesic distance from ***x*** to its *k* nearest neighbors. Other geodesic distances are found from matrix $\overset{˜}{D}$ by computing the shortest paths as it has been done while training the model. If value $\frac{d^{2}\left(x,x_{i}\right)\;}{\beta }$ is too large (which happens when average distances between compounds in the original data space are much exceed one), additional coefficient *β*_1_ can be used for both training the model and extending it to the new points. In this case the parameter *β* in Eq. 11 is replaced with *β*_1_*β*.

### Applicability domain approaches

#### ***Ball***

Ball [50] is a distance-based method for outlier detection. It uses ***L***^*p*^ –metric, in which distance between compounds ***x*** and ***y*** in feature is space denoted by Formula 11.

(12) distLpx,y=∑ixi-yip1p

The algorithm optimizes the weight vector ***w*** the following way:

(13) $$\begin{array}{c} \text{min}\rho \\ s.t.\underset{j}{\sum }w_{j}{\left|x_{\mathrm{ij}}-a_{j}\right|}^{p}\leq \rho \\ \underset{j}{\sum }w_{j}=1,w_{j}\geq 0 \end{array}$$

where ***α*** is a centroid of the data points and *x*_*ij*_ denotes the coordinate *j* of the compound ***x***_*i*_.

**Figure 1:**
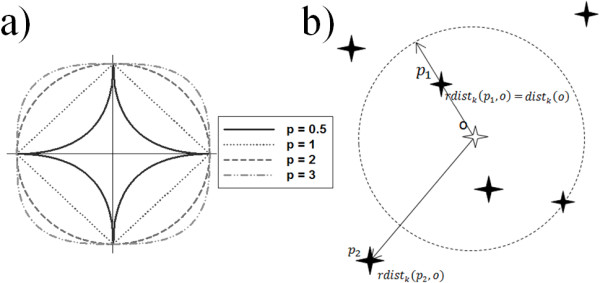
**The methods of applicability domain estimation: a) The *L***^***p***^**“ball” of radius one in 2-dimentional space for different values of *p *and *w***_**1**_ **=** ***w***_**2**_**; b) the reachability distance of objects *p***_**1 **_**and *p***_**2 **_**with respect to object *o *for *k*** **= 3.**

After ***w*** is optimized, the compounds ***x***_*i*_ for which $\underset{j}{\sum }w_{j}{\left|x_{\mathrm{ij}}-a_{j}\right|}^{p}$ is the largest are considered as outliers. In other words this method fit ***L***^*p*^ “ball” around the data. This “ball” separates targets from outliers. Figure 1a demonstrates the case of 2-dimensional feature space with *w*_1_ = *w*_2_.

#### ***Local Outlier Factor (LOF)***

LOF is a probability based method for outlier detection in a multidimensional dataset [51]. It operates with local densities of objects in the dataset by using the definition of local reachability density and calculates value of “local outlier factor” that indicates the degree of object’s dissimilarity to other compounds in the data set.

To define the local reachability density we should first introduce some other concepts. We call *k*-distance of the object *p* (*dist*_*k*_(*p*)) the smallest value for which there are at least *k* objects besides *p* with a distance from *p* smaller or equal to *dist*_*k*_(*p*). *K*-distance neighborhood of an object *p* (*N*_*k*_(*p*)) is a set of objects, not including *p*, whose distance from *p* does not exceed *dist*_*k*_(*p*). Let us specify that the cardinality of *N*_*k*_(*p*), which we also denote as |*N*_*k*_(*p*)|, can be greater than *k* in case, when in *N*_*k*_(*p*) exist two or more objects whose distances from *p* are equal to *dist*_*k*_(*p*). Reachability distance of object *p* with respect to object *o* (*rdist*_*k*_(*p,o*)) is the maximum value between *k*-distance of *o* and distance from *o* to *p*. The idea of reachability distance is illustrated in Figure 1b.

Local reachability density can be defined as

(14) $$\mathrm{lr}d_{k}\left(p\right)={\left(\frac{\sum_{o\in N_{k}\left(p\right)}\text{rdis}t_{k}\left(p,o\right)}{\left|N_{k}\left(p\right)\right|}\right)}^{-1}$$

The local outlier factor is an average of the ratios of the local reachability densities of objects to those of object’s *k* nearest neighbors (Eq. 15).

(15) $$\mathrm{LO}F_{k}\left(p\right)=\frac{\sum_{o\in N_{k}\left(p\right)}\frac{\mathrm{lr}d_{k}\left(o\right)}{\mathrm{lr}d_{k}\left(p\right)}}{\left|N_{k}\left(p\right)\right|}$$

In [51] is shown, that LOF of objects that lie ‘deep’ inside a cluster approximately equals to 1. It is also shown that in majority of cases k can be chosen so that for all objects that belong to some cluster of objects LOF approximately equals to one, and for any other object it significantly differs from one. This fact allows us to detect compounds that do not belong to any cluster and so can be called outliers.

## Experimental

The predictive performance of developed classification models was assessed using five-fold external cross-validation (5-CV) procedure considering Balanced Accuracy (BA) value [79] as a criterion of the predictive performance of the models. BA is an average of two other criteria, Sensitivity and Specificity, which were designed to assess model’s ability to identify compounds from a certain class (active or positive for Sensitivity and inactive or negative for Specificity) disregarding its behavior for the other class. The combination of Sensitivity and Specificity should be able to compensate possible imbalance in the dataset.

(16) $$\mathrm{BA}=\frac{1}{2}\left(\text{Sens}+\text{Spec}\right)=\frac{1}{2}\left(\frac{\mathrm{tp}}{\mathrm{tp}+\mathrm{fn}}+\frac{\mathrm{tn}}{\mathrm{tn}+\mathrm{fp}}\right)$$

where *Sens* is Sensitivity, *Spec* is Specificity, *tp* stands for *true positive* rate (e.g. the number of correctly predicted active compounds), *tn* – for *true negative* (correctly predicted inactive compounds), *fp* – for *false positive* (inactive compounds that’ve been predicted to be active), *fn* – for *false negative* (active compounds that’ve been identified as inactive ones).

LibSVM [80] was used for developing SVM models, two its external parameters *v* and *γ* were varied from 0.01 to 0.91 and from 2^-11^ to 2^3^ respectively.

GTM models were built with the help of the Netlab [81] package. This implementation can’t work with large number of descriptors, so the Principal Component Analysis was introduced beforehand. Here, the following external parameters were gone over. Number of first principal components, that were retained, was varied from 20 to 60, number of latent points – from 5^2^ to 50^2^, number of radial basis network centers – from 2^2^ to 7^2^.

PNN was implemented in Classification Toolbox for use with MATLAB [82]. Its only external parameter Gaussian width was chosen from the range of [0; 1].

Among all the developed models for each combination of dataset, descriptor type and applied method one with the highest Balanced Accuracy was selected for further analysis.

For s-GTM the value of threshold was tried from 0 to 0.2, in most cases we used *ρ* = 40 or *ρ* = 30. The only external parameter in the movement step (responsibility radius, *rr*) has a great influence on the model. Too small values leads to small changes in the model compared to unsupervised GTM, too big – to mapping all compounds into a single point. This parameter was sorted out in a large range.

The performance of data visualization has been monitored with three quantitative measures. Each of them is normalized to vary from 0 to 1 and can be computed for a data set where the information about the classes is available.

### Г-score

Г-score [26] takes into account *k* nearest neighbors of each projection. The more neighbors of each point belong to the same class, the higher is Г-score. Thus, this score characterizes the ability of a model to produce similar-structure clustering in a visualization. To compute Г-score one need to take the following steps. First, for each compound *v*_*l*_*G*(*l, k*) should be computed:

(17) $$G\left(l,k\right)=\frac{1}{k}\overset{k}{\underset{j=1}{\sum }}g\left(\nu_{l},j\right)$$

where *k* is the number of nearest neighbors, which is an external parameter, *g*(*ν*_*l*_, *j*) = 1 if the *j*th nearest neighbor of *v*_*l*_ in the visualization space belongs to the same class as *v*_*l*_, g (*v*_*l*_*,j*) = 0 otherwise. Then for each class *i γ*_*i*_ (*k*) is defined as

(18) $$\gamma_{i}\left(k\right)=\frac{1}{n_{i}}\overset{n_{i}}{\underset{l=1}{\sum }}G\left(l,k\right)$$

where *n*_*i*_ is a number of compounds of class *i*. And finally the Г-score is

(19) $$\Gamma \left(k\right)=\frac{1}{N}\overset{N}{\underset{i=1}{\sum }}\gamma_{i}\left(k\right)$$

where *N* is a number of classes.

### Distance Consistency (DSC)

DSC [83] is based on the distances from points to the centroid of each class. It is higher when more points are closer to the centroid of the corresponding class, then to any other. The score is equal to 1, if the model projects compounds into separate clusters, one for each class. The computation of DSC is similar to the computation of Г-score, but instead of *g*(*v*_*l*_*, j*) the centroid distance (CD) is used. Beforehand for each class *i* one need to find the coordinates of its centroid *c*_*i*_. Then *CD*(*ν*_*l*_, *c*_*i*_) = 1 if the closest to *v*_*l*_ is the centroid *c*_*i*_ and *v*_*l*_ belongs to class *i* and *CD*(*ν*_*l*_, *c*_*i*_) = 0 otherwise. Then for each class *i*

(20) $$C\left(i\right)=\frac{1}{n_{i}}\overset{n_{i}}{\underset{l=1}{\sum }}\mathrm{CD}\left(\nu_{l},c_{i}\right)$$

(21) $$\text{DSC}=\frac{1}{N}\overset{N}{\underset{i=1}{\sum }}C_{i}$$

### Distribution Consistency (DC)

DC [83] estimates the overlapping of classes. It divides a map into separate areas and treats them independently. For each area the value of entropy is computed, which is 0, if all the points in the area share one class label, and reaches maximum, when every class is represented in the area by equal number of points. For DC computation the conception of entropy of the region *R* is to be introduced.

(22) $$H_{R}=-\overset{N}{\underset{i=1}{\sum }}\frac{p_{i}}{\sum_{i}p_{i}}{\text{log}}_{2}\left(\frac{p_{i}}{\sum_{i}p_{i}}\right)$$

Here, *p*_*i*_ is a number of molecules of class *i* in the region *R*. And the value of DC is defined the following way

(23) $$\mathrm{DC}=1-\frac{1}{Z}\underset{R}{\sum }p_{R}H_{R}$$

*p*_*R*_ is the whole number of molecules in the region R and a coefficient *Z* = *n* log_2_*N* is used to range DC from 0 to 1. In this work to obtain the required regions we divided the visualization map into 15 × 15 equal sized rectangles.

## Results and discussion

### Classification models performance

**Figure 2:**
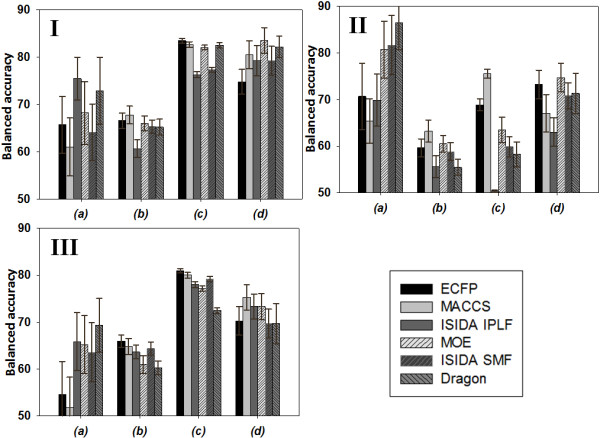
**Predictive performance (*Balanced Accuracy*) of SVM *(I)*, GTM *(II) *** **and PNN *(III) *** **approaches for different types of descriptors and different datasets: acute toxicity *(a)*, carcinogenicity *(b)*, mutagenicity *(c)*, phospholipidisis *(d)*.**

As one can see in Figure 2, for three out of four considered datasets, the best predictive performance was demonstrated by the Support Vector Machine approach (carcinogenicity – 68%, mutagenicity – 83%, phospholipidosis – 82%). Yet, in prediction of acute toxicity GTM significantly outperformed SVM (Balanced Accuracy reached 86% for GTM and 75% for SVM models). It is also seen that for GTM approach IPLF descriptors shown to be less effective than others, while applying molecular fingerprints for both SVM and GTM approaches led to high values of Balanced Accuracy. The behavior of accuracy for acute toxicity predictions significantly differs from those for other data sets. Molecular fingerprints here showed nearly the worst results among all the types of descriptors in this study (63% for SVM and 71% for GTM), while the best predictive performance was achieved using descriptors of the MOE and Dragon packages. The corroboration and a possible explanation of this fact may be given by found in the attempt of detection of structural alerts that is given further in this chapter. Implementation of MACCS descriptors failed to mark out any fragments that are responsible for toxic activity of compounds. The reason for this may be the imbalance of this particular data set. The deficiency of inactive compounds leads to difficulties in determining whether the presence of a fragment in several inactive compounds is an accident. Though in most cases SVM outperforms GTM, the analysis of its work is obstructed by the lack of intrinsic information about the predictive decisions. GTM, on the other hand, not only gives easily interpretable probability distribution for each compound, but also can be used as a tool for data visualization and outlier detection.

PNN may be considered a compromise between the lack of method’s internal information of SVM and the decrease of accuracy of GTM. It is not such a universal tool as GTM but slightly outperforms it (up to 6% for mutagenicity). At the same time, PNN makes less accurate predictions then SVM, but allows one to look through the background of each decision by analyzing pattern and decision layers. There is a similarity in behavior of SVM and PNN.

Dependence of Balanced Accuracy from datasets and descriptor types obtained by PNN is turned out to be similar to that of SVM, but not of GTM, though both PNN and GTM are neural networks.

The considered data sets were previously studied by other teams. Thus, classification of the acute toxicity data set has been performed in [84]. The compounds have been divided into classes differently than in our study and in the original database. A set of different machine learning approaches including several types of neural networks as well as SVM, Decision Trees and Gene Expression Programming have been applied for classification purposes. Corresponding Balanced Accuracy values of the developed models varied in the range from 0.85 to 0.93. A number of studies [85,86] with the regression analysis have been published including the original publication of this data set [61]. The carcinogenicity data involved in this study has been used in QSAR studies mostly as a source of further data retrieval. It has been used, for example, as a part of considered data in [87]. A thorough analysis of the mutagenicity data set including the applicability domain estimation has been performed in [40]. The direct comparison of the obtained results performance is straitened because of the difference in the statistical parameters used. Comparable results (obtained by combination of ECFP descriptors with Random Forest and Nearest Neighbor classifiers) have been recently reported in [88]. In [59] SVM and Random Forest were applied for phospholipidosis prediction. There Matthews Correlation Coefficient was used to assess the results performance, and its values varied up to 0.72 that outperforms the maximum value of this parameter in our study.

**Figure 3:**
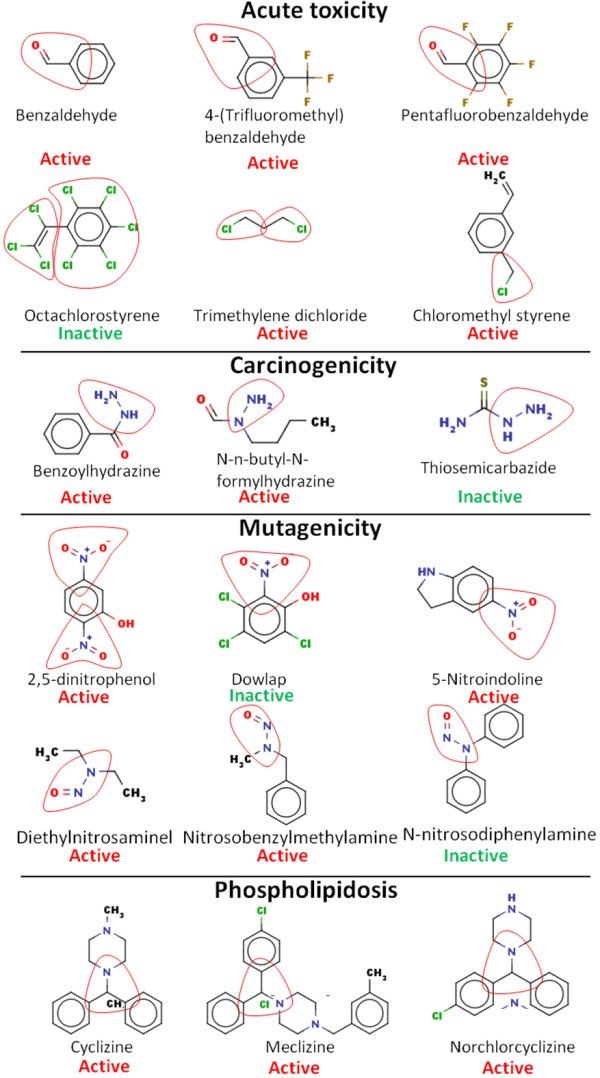
**Examples of the descriptors frequently used to predict compounds as active by all three applied methods.** For each descriptor an example of inactive compound is given (if any).

Predictions of models developed on IPLF, ECFP and SMF descriptors were analyzed. The numbers of compounds containing a certain descriptor *d*, predicted to be active $n_{\text{act}}^{d}$ and inactive $n_{\text{inact}}^{d}$, were calculated for each descriptor. Then the corresponding fractions of compounds were calculated as

(24) $$fr_{\text{act}}^{d}=\frac{n_{\text{act}}^{d}}{n_{\text{act}}},fr_{\text{inact}}^{d}=\frac{n_{\text{inact}}^{d}}{n_{\text{inact}}}$$

where *n*_*act*_ and *n*_*inact*_ are total number of compounds, predicted to be active or, respectively, inactive, by the model at issue. The rare descriptors with $fr_{\text{act}}^{d}+fr_{\text{inact}}^{d}<0.05$ were excluded from further consideration. Among other descriptors were selected for further analysis those with $\frac{fr_{\text{act}}}{fr_{\text{inact}}}>2.5$. Fragment descriptors used for the prediction of compounds as actives by all three classification methods are demonstrated in Figure 3.

MACCS descriptors were not effective in detecting structural alerts for all data sets, but mutagenicity, where eight descriptors detected mostly nitro groups. There are limited number of descriptors, which all three methods considered to be structural alerts. PNN tends to attribute descriptors to structural alerts that may be one of the reasons of its inferior efficiency compared to SVM. The described approach didn’t allow detecting structural alerts for phospholipidosis. Though more than 30 descriptors were unanimously marked by the methods, all these descriptors refer to several groups of active compounds with similar structure (an example is demonstrated in Figure 3).

### Performance of data visualization models

In this study, supervised extensions of Isomap and GTM were used for data visualization.

**Figure 4:**
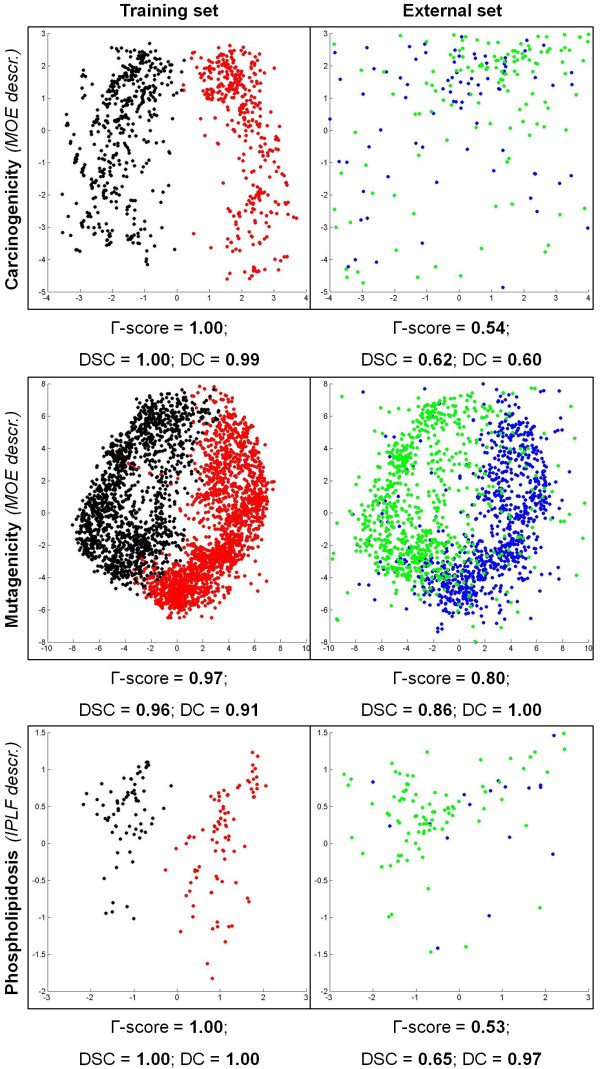
**S-Isomap data visualization for considered datasets (the maps for the best combination of involved approach and descriptor type are given).** Each point in the map corresponds to the individual compound (in red, blue - actives, black, green - inactives). In the left column the values of visualization quality assessment parameters are presented for the training set, in the right one – for the test set.

**Figure 5:**
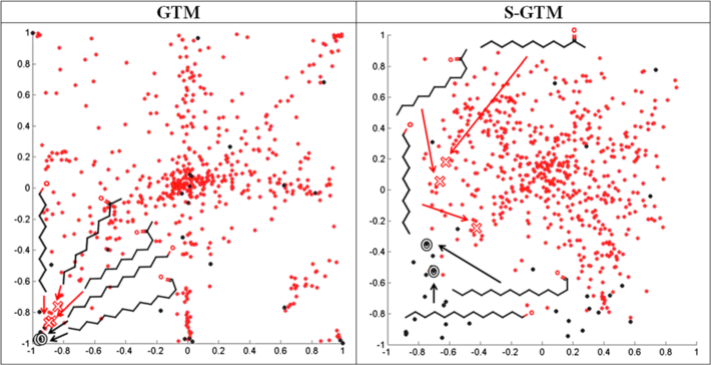
**The visualization maps of acute toxicity dataset (MOE descriptors), obtained by unsupervised (left) and supervised (right) GTM.** The singled out molecules share similar structure but belong to different classes. They are mapped close to each other by GTM, but are distinguishable in the s-GTM visualization.

**Figure 6:**
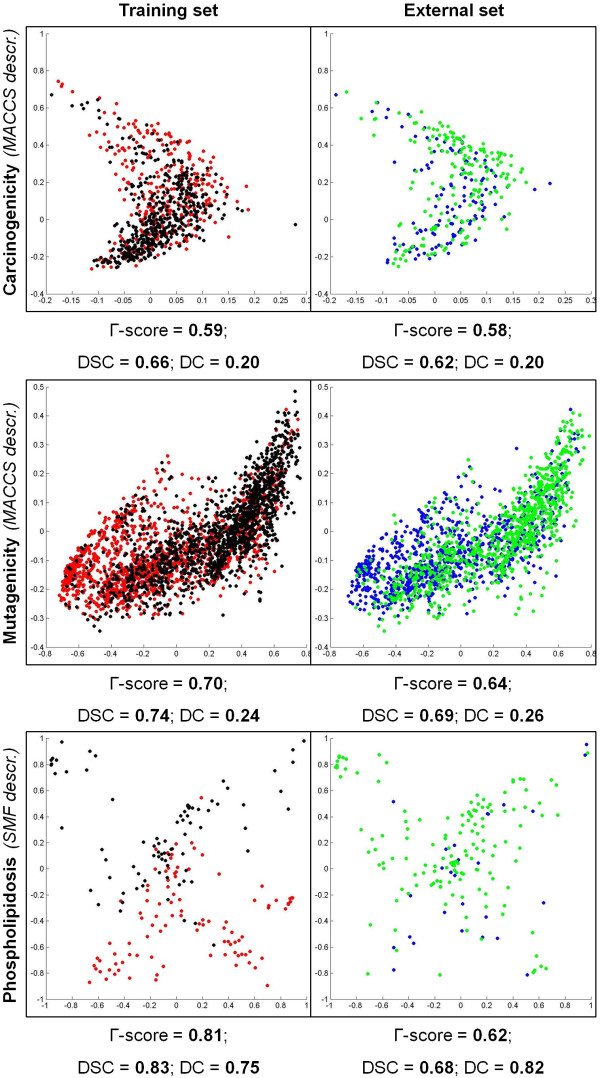
**S-GTM data visualization for considered datasets (the maps for the best combination of involved approach and descriptor type are given).** Each point in the map corresponds to the individual compound (in red, blue - actives, black, green - inactives). In the left column the values of visualization quality assessment parameters are presented for the training set, in the right one – for the test set.

S-Isomap was first introduced in [53]. It demonstrated excellent results in separation different classes of training set. Mapping of the external test set is an important part of the chemography from the practical point of view in the context of the possibility of the application of the developed models to virtual screening and to mechanistic model interpretation which allows one to understand which changes of the existing structures are required to improve target properties, to generate new hypothesis and, finally, to optimize the chemical structures. In the original article for mapping an external test set it was recommended to use Radial Basis Network. In our study it turned out to be ineffective for diverse sets of chemical compounds. In this study, we propose new approach for the application of models to visualizing external data. We modified an approach proposed in [77,78] to adapt it for s-Isomap (See details in Method’s description). The results of the mapping of external test sets for three types of activities are demonstrated in Figure 4. Hereinafter visualization maps are presented in the coordinate system generated be the applied methods. GTM and s-GTM presume that latent space is a rectangle of size 2 × 2 with its center located at (0, 0). Isomap and s-Isomap project compounds into two-dimensional space so that Euclidean distance (for Isomap) or dissimilarity measure (see Eq. 10) (for s-Isomap) can be preserved and the map scale is chosen accordingly. All axes in Figures 4, 5 and 6 are relative and have no units of measurement.

One can see that while s-Isomap performed almost perfect separation of the training set (none of the applied assessment parameters decreased below 0.91), the quality of mapping an external set for these models is highly dependent on the dataset in consideration. An external set of mutagenicity was mapped quite accurate (Г-score = 0.80, DSC = 0.86, DC = 1.00), while the mapping of external set for carcinogenicity is moderate: the corresponding parameters varied in the range 0.54-0.62. One of the main factors that determine the quality of mapping is the distance from each point of the external set to the nearest neighbors in the training set. The closer they are, the better are the results. In case, if the distances are much greater than one, but are of the same scale, the additional parameter *β*_1_ can be used to put them to the desirable range (look [53] for the specific values). In the case of carcinogenicity, in particular, the distances from the points of the external set differ for several degrees.

The supervised extension of GTM is proposed in this paper for the first time. It demonstrates a significant improvement in visualization performance. An example for acute toxicity dataset and MOE descriptors is given in Figure 5. Besides a noticeable increase in all three used visualization quality measures (Г-score raised from 0.62 for unsupervised model to 0.77 for the supervised one, DSC – from 0.57 to 0.87 and DC – from 0.85 to 0.95, respectively), one can see how structurally similar compounds related to different classes and close to each other on the map obtained by unsupervised GTM are separated using supervised extension of GTM. Here, two groups were selected, each of them contained structurally similar active and inactive compounds. The first one contains toxic 1-Decanol and non-toxic 1-Tridecanol that differ from each other only by the length of the carbon chain (Tanimoto Similarity Coefficient (TSC) is equal to 1.00). The second group consists of toxic 2-Undecanone and 2-Dodecanone and similar to them (TSC = 0.82) non-toxic 3-Tetradecanal. All these compounds were mapped into a small area by unsupervised GTM while well distinguished applying its supervised extension.

Mapping of external test set for s-GTM is performed using the same procedure as for GTM, and the corresponding results are demonstrated in Figure 6. One can see that presented visualization maps are inferior to those of s-Isomap. At the same time s-GTM performs more accurate mapping of the external test set than s-Isomap, since after the model has been trained, the training set is mapped using the same algorithm as is used for the mapping of an external test set. In s-GTM, if one includes a compound from the training set in the test set, it will projected exactly to the same point of the map. This is not so for s-Isomap. Without label information each mapping will be an approximation and can be performed in different ways. The one we’ve proposed is based on the assumption that label information does not have much influence on the relative location of the points that are close to each other. During the training process s-Isomap changes distances between compounds in different manners regarding if the compounds belong to the same class or not but proportionally their relative position. Thus, new distances for compounds from different classes do not change significantly if they are close to each other. And if the compound from the test set has close neighbors in the training set, they will mapped close even if they belong to different classes. In Figure 6, as well as in Figure 4, acute toxicity maps are not presented since we had no corresponding external set at our disposal. Nevertheless, s-GTM demonstrated reasonably high results visualizing this data set. Considered quantitative measures for the best maps varied in the following ranges as a function of the descriptors type: Г-Score – 0.76-0.77; DSC – 0.72-0.87; DC – 0.93-0.96.

The given examples allows one to assume that s-GTM tends to form clusters of identically labeled projections that is reflected by the increase of the DSC value as compared with the results of original GTM. For instance, for presented in Figure 6 examples the improvement in DSC is 0.12 for carcinogenicity, 0.18 for mutagenicity and 0.21 for phospholipidosis. At the same time, while generally s-GTM provides at least slight increase in all the considered parameters for visualization quality assessment, it doesn’t separates areas of overlapping as successfully as s-Isomap does. The reason for this is that s-GTM works with the given relative location of compounds in the data space, while s-Isomap changes the distance between the compounds according to the label information (and thus performs some sort of metric learning [89]). E.g. if the choice of descriptors leads to overlapping differently labeled compounds in the original data space, s-GTM may not be able to separate them completely, but will project an external set following the pattern of the training set, while s-Isomap can achieve almost perfect separation for the most difficult visualization tasks, but then one may face some problems with the mapping of the external set.

For each presented map (Figures 4, 5 and 6) the values of three quantitative measures of visualization performances are given. None of the parameters is perfect and can be individually applied for identification of adequate data visualization models and comparison of different maps. Г-score, for example, is high for the maps with randomly mixed compounds that are still grouped in small clusters. Distance consistency can be low for well separated classes that form non-convex figures. Distribution Consistency is usually high for imbalanced dataset visualization and strongly depends on its external parameter. The effectiveness of each parameter is defined by the nature of obtained map. For example, the maps may have similar DC value, but differ in DCS, which can be interpreted that considered maps have similar class overlapping and different level of clusterization. In this study, the combination of DC and DSC parameters demonstrates its performance. Another advantage of DC and DSC is its less time- and memory-consuming compared to Г-score.

### Applicability domain of models

Two methods of applicability domain estimation were applied in this study, their performance was compared. One of them is a distance-based Ball, the other – a distribution-based LOF. The Principal Component Analysis was used as a pre-processing step. Each method was used to generate a sorted list of compounds according to their “outlierness” (the value of LOF function for LOF and distance to the centroid for ball). The impact of outliers’ exclusion on the Balanced Accuracy of the models was analyzed.

**Figure 7:**
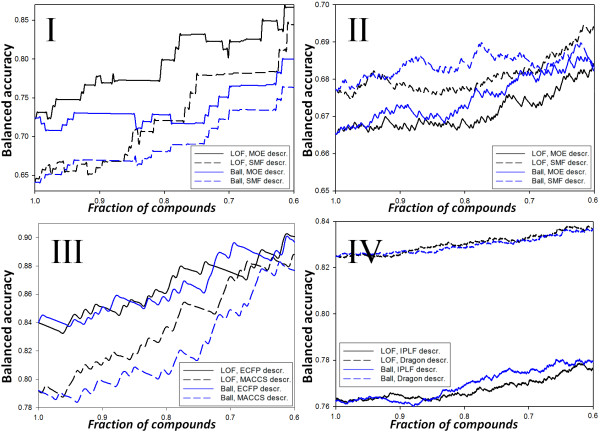
**Balanced accuracy for SVM models as a function of data fraction.** The compounds were removed according to the rate of their dissimilarity from the rest of the training set, which was assessed by the applicability domain methods. I – acute toxicity, II – carcinogenicity, III – phospholipidosis, IV - mutagenicity.

In Figure 7 the Balanced Accuracy is given as a function of data fraction after the exclusion of outliers. The nature of the changes is affected by the distribution of compounds between classes in the dataset and predicting performance. In all the presented cases one can see a certain growth in performance which is different for considered datasets. Thus, the Balanced Accuracy of models for predicting carcinogenicity has increased only by 2.5% and after almost half of the compounds has been removed, while application of LOF to the SVM model for phospholipidosis that used SMF descriptors yield almost linear growth of Balanced Accuracy from 79% to 88% (after excluding 0.4 of compounds).

For acute toxicity LOF proved to be more efficient than *Ball*. This can be explained by the presence of several clusters with high density of compounds in the dataset containing compounds of different classes. The compounds in these clusters may have been correctly classified, while a number of false predictions were made for the compounds lying in the areas of classes overlapping in the midst of the clusters. In this case LOF was able to detect these mispredicted compounds as outliers and *Ball* just excluded the most distant from the centroid compounds in spite of the density distribution.

For phospholipidosis *Ball* and LOF demonstrated similar performance, though LOF is a bit more efficient. It may indicate that the data are slightly clusterized with an area of clusters’ overlapping and most incorrectly predicted compounds are located far from the main aggregation of the chemical structures.

For carcinogenicity both applied methods demonstrated only a small increase of the Balanced Accuracy, with a better performance of *Ball* (in Figure 7 blue lines lie above corresponding black lines). This could happen if the projection of the dataset into the data space was a one cluster with irregular density distribution and large area of classes overlapping.

Similar pattern can be found for mutagenicity. Here, the maximum increase in BA is only about 2% and *Ball* only slightly outperforms LOF for IPLF descriptors. In respect with reasonable performances of both visualization and classification methods for mutagenicity dataset, one may assume that this dataset doesn’t contain many outliers and applying applicability domain analysis does not affect the predictive performance of models.

**Figure 8:**
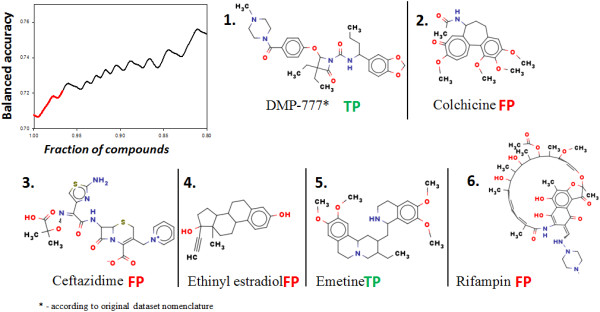
**The examples of outliers, identified by LOF for combination of phospholipidosis and SMF descriptors and the influence of their exclusion on the Balanced Accuracy of a corresponding GTM model.** The compounds given as examples are the ones that were marked as the most distant from the applicability domain. The red area on the diagram reflects the influence of their exclusion on the Balanced Accuracy of the model.

To demonstrate the principles of the outlier detection, the example of the compounds marked by LOF as the most dissimilar to the rest of the phospholipidosis data set was provided in Figure 8. The diagram on the upper left corner illustrates the effect of their exclusion on the Balanced Accuracy of the model obtained by GTM.

The SMF descriptors we used represent only terminal groups (See the section devoted to the descriptor types). The presented compounds were considered as outliers not because of the presence of some unique fragment, but because of unique or rare combination of atoms and bonds and their relative location. For example, Ceftazidime is the only compound in the dataset that contains sulfur with aromatic bond together with distanced heteroatoms (from 9 to 15 atoms in a fragment). And only in Rifampin there are carbon atoms with double bonds having from 4 to 10 atoms between them. Not all the given compounds are characterized by a number of unique descriptors, but all of them contain plenty of rare ones, as, for example, Colchicine.

## Conclusions

This work concerns an approach that combines several classification and chemography methods for *in silico* assessment of chemical liabilities and for the interpretation of obtained results in the context of impact of structural changes of compounds on their pharmacological profile. Support Vector Machines, Generative Topographic Mapping and Probabilistic Neural Network were used for classification. The classification performances were improved by combination with two applicability domain assessment approaches (Ball and Local Outlier Factor), and their contribution was analyzed. Here, the supervised extension of Generative Topographic Mapping was proposed as new efficient chemography method. New approach for mapping new data using supervised Isomap without re-building models from the scratch has been proposed. The evaluation of the performance of the dimensionality reduction techniques and introduced descriptor spaces to separate different activity classes has been monitored by three parameters (Г-score, Distance Consistency and Distribution Consistency) and their efficiency was compared. The obtained results, which are comparable with or exceed those, published by other teams for the given biological activities, allow one to use proposed approach as an efficient filter for exclusion of compounds with undesirable activities on early stages of drug design process.

## Competing interests

The authors declare that they have no competing interests.

## Authors’ contributions

AT provided regular supervisory input throughout the course of this work. NK conceived of the study, participated in its design, carried out calculations, and drafted the manuscript. SO participated in its design, carried out calculations, and drafted the manuscript. AB and ED carried out calculations. All authors read and approved the final manuscript.
